# Plasma oxidized high-density lipoprotein and glycated apolipoprotein A-I concentrations in ST-segment elevation myocardial infarction patients with stress hyperglycaemia or high thrombus burden

**DOI:** 10.1080/03009734.2018.1494229

**Published:** 2018-09-27

**Authors:** Bing-Qiang Li, Yu-Cheng Zhong, Xiang Wang

**Affiliations:** a The First Affiliated Hospital, and College of Clinical Medicine, Henan University of Science and Technology, Luoyang, P. R. China;; b Department of Cardiology, Union Hospital, Tongji Medical College, Huazhong University of Science and Technology, Wuhan, P.R. China

**Keywords:** Glycated apolipoprotein A-I, high thrombus burden, oxidized high-density lipoprotein, stress hyperglycaemia, ST-segment elevation myocardial infarction

## Abstract

**Background:** High-density lipoprotein (HDL) particles exert many beneficial actions that may help protect against cardiovascular disease. However, recent work has demonstrated that HDL can be oxidized and glycated under certain circumstances and may become pro-atherogenic. The present study investigated the impact of oxidized high-density lipoprotein (ox-HDL) and glycated apolipoprotein A-I (gly-ApoA-I) in patients presenting with ST-elevation myocardial infarction (STEMI).

**Methods:** We assessed 55 consecutive patients with STEMI. Patients were divided into: (1) a stress hyperglycaemia (SH) and a no SH group; and (2) a high thrombus burden (HTB) group and a low thrombus burden (LTB) group. Meanwhile, 48 healthy volunteers were recruited as controls. Plasma ox-HDL and gly-ApoA-I concentrations were measured on admission and 7 days after admission.

**Results:** Higher concentrations of ox-HDL and gly-ApoA-I were found in the STEMI group than in the control group on admission and at d7. Further subgroup analysis showed that ox-HDL and gly-ApoA-I were higher in the SH group than in the no SH group at both time points; the HTB group had higher ox-HDL and ox-HDL/HDL-C levels than the LTB group on admission and at d7. However, gly-ApoA-I and the relative intensity of ApoA-I glycation showed no significant differences between the HTB and LTB groups.

**Conclusions:** The present data indicate that: (1) SH is associated with increased plasma concentrations of ox-HDL and gly-ApoA-I and therefore aggressive treatment is recommended; and (2) that ox-HDL and ox-HDL/HDL-C were higher in the HTB group and may be used to quantify thrombus burden.

## Introduction

High-density lipoprotein (HDL) is a plasma protein that is primarily synthesized in the liver and small intestine. Apolipoprotein A-I (ApoA-I) is the main protein in HDL that plays a crucial role in the first cholesterol transport reversal step by enhancing the sterol efflux from macrophages (1). HDL is an anti-atherogenic lipoprotein; a low plasma concentration of HDL-cholesterol (HDL-C) is a risk factor for coronary heart disease (CHD) (2). However, recent research has shown that HDL and/or ApoA-I are susceptible to structural modifications mediated by various mechanisms, including oxidation and glycation. Myeloperoxidase, superoxide anion radical (O_2_^−^), and H_2_O_2_, secreted by activated phagocytes, may be potential candidates for the generation of oxidized HDL (ox-HDL) *in vivo* (3). It has been demonstrated that glucose or arginine induced a nucleophilic non-enzymatic glycation of ApoA-I to form glycated ApoA-I (gly-ApoA-I) (4,5). HDL becomes dysfunctional when it is oxidized or glycated (6). Plasma concentrations of ox-HDL in diabetic patients and young myocardial infarction patients have been shown to be significantly higher than in healthy people (7). Moreover, glycation of apoprotein A-I was found to be associated with coronary artery plaque progression in type 2 diabetic patients (8). These studies demonstrated that the increase of these structural modifications may play important roles in the pathogenesis of CHD.

ST-elevation myocardial infarction (STEMI) is the most serious kind of CHD. Stress hyperglycaemia (SH) and high thrombus burden (HTB) are common manifestations of STEMI. It has been reported that hyperglycaemia causes oxidative stress, enhances inflammation, induces apoptosis, and activates coagulation, deteriorating the myocardial damage in the setting of ischaemia (9–11). Therefore, HDL is easily oxidized or glycated in STEMI patients. Ox-HDL and gly-ApoA-I lose their atheroprotective properties and may promote thrombosis. At present, little is known about the concentrations of ox-HDL and gly-ApoA-I in STEMI patients and their clinical relevance. In the present study, we measured levels of ox-HDL and gly-ApoA-I in STEMI patients and grouped patients according to their plasma glucose concentration on admission or their thrombus burden.

## Materials and methods

### Patients

We recruited consecutive STEMI patients who underwent primary percutaneous coronary intervention (PCI) between June 2015 and December 2015 in the Union Hospital of Huazhong University of Science and Technology, Wuhan, China. Fifty-five eligible patients were enrolled in the STEMI group. STEMI patients were divided into: (1) a SH group with 28 patients, and a no SH group with 27 patients, depending on blood glucose concentrations on admission; and (2) a HTB group with 26 patients and a low thrombus burden (LTB) group with 29 patients, depending on the thrombus burden. Simultaneously, we recruited 48 healthy volunteers as controls. Inclusion criteria for the STEMI group were as follows: myocardial infarction confirmed by a significant rise of cTNI and creatinine kinase MB (CK-MB) levels, ST-segment changes, and a duration of chest pain >30 min on admission within 12 h.

Inclusion criteria for the control group: healthy normolipidaemic volunteers with normal cardiac function and no other cardiovascular risk factors. No subjects were treated with drugs such as β-blockers, angiotensin-converting enzyme inhibitors (ACEI)/angiotensin receptor blockers (ARB), statins, antiplatelet agents, or calcium channel blockers.

Exclusion criteria were as follows: (1) acute or chronic infectious diseases or autoimmune diseases; (2) cardiomyopathy and heart valve disease; 3) liver disease, renal insufficiency, gastrointestinal diseases, and diseases of the blood; 4) thyroid dysfunction and tumour; 5) surgery or trauma; and 6) a history of myocardial infarction and PCI.

Stress hyperglycaemia was defined as blood glucose ≥10 mmol/L (180 mg/dL) on admission, regardless of whether the patient had a history of diabetes or not (12). Intracoronary thrombus was angiographically identified (only STEMI patients) and was scored in six grades (13): grade 0 (G0), no detectable thrombus; grade 1 (G1), features suggestive but not definitive for thrombus (reduced contrast density, haziness, irregular lesion contour, or a smooth convex meniscus at the site of occlusion); grade 2 (G2), definite thrombus with greatest dimensions ≤0.5 of vessel diameter; grade 3 (G3), definite thrombus with greatest linear dimension >0.5 but <2 vessel diameters; grade 4 (G4), definite thrombus with greatest linear dimension ≥2 vessel diameters; and grade 5 (G5), complete and total occlusion with the inability to assess the thrombus dimensions. We further stratified patients into two groups: LTB (G0–G3) and HTB (G4–G5).

### Blood samples

In the STEMI group, blood samples were obtained on admission (immediately after arrival at the coronary care unit) and 7 days after admission (after overnight fasting). In the control group, blood samples were obtained after overnight fasting. Each blood sample was divided into two parts. One was used for measurements of relevant biochemical and immune parameters, and the other was immediately used for separation of the HDL. Blood glucose, high-density lipoprotein cholesterol, low-density lipoprotein cholesterol, serum apolipoprotein A-I, serum creatinine, CRP, BNP, and cTNI levels were measured at the clinical laboratory of the Union Hospital of Huazhong University of Science and Technology.

### ELISA

Plasma concentrations of ox-HDL (OxiSelect™ Human Oxidized HDL ELISA Kit, Cell Biolabs, Inc.) were measured by an enzyme-linked immunosorbent assay (ELISA), following the manufacturer’s instructions. The minimal detectable concentration was 1 ng/mL for ox-HDL. All samples were measured in duplicate. Then, we calculated the ox-HDL/HDL-C.

### Antibodies and Western blot analysis

Isolated HDL proteins were separated on SDS-polyacrylamide gels and then transferred to polyvinylidene fluoride membranes. After blocking with 5% milk, the membranes were incubated overnight at 4 °C with anti-ApoA-I (RD system) or an anti-N´-(carboxymethyl)-lysine (CML) antibody (RD system). Immobilon™ Western ChemilumInescent HRP Substrate (Antagene Kit No. ANT038) was used after reaction with a secondary antibody. Images were analysed and quantified using Adobe Photoshop CS2 software. A band of 28 kDa was validated to be ApoA-I protein by Western blot, and Western blot was also used to measure the glycation level of ApoA-I protein in STEMI and control groups, using anti-CML antibody. Absolute intensity was calculated by multiplying the mean density value by pixel for each band. Relative intensity of ApoA-I glycation was calculated as the absolute intensity of ApoA-I glycation divided by that of protein ApoA-I.

### Doppler echocardiography

Patients underwent M-mode and 2D-echocardiography using a GEViVidE7 ultrasonography machine (GE Healthcare, USA) with a transthoracic 1.5–4.3 MHz probe (M5S-D). Left ventricular end-diastolic diameter (LVEDD) and fractional shortening were measured. Left ventricular ejection fraction (LVEF) was calculated from the apical four-chamber position by the area-length method. In addition, echocardiography was performed within 6 h after patient arrival in the CCU.

### The Gensini score

The severity of coronary stenosis in patients was estimated by the Gensini coronary score following coronary angiography. The Gensini score was computed by assigning a severity score to each coronary stenosis according to the degree of luminal narrowing and its geographic importance. Reduction in the lumen diameter and the roentgenographic appearance of concentric lesions and eccentric plaques were evaluated (reductions of 25%, 50%, 75%, 90%, and 99% and complete occlusion were assigned Gensini scores of 1, 2, 4, 8, 16, and 32, respectively). The score was then multiplied by a factor that incorporated the importance of the lesion’s position in the coronary arterial tree as follows: 5 for the left main coronary artery; 2.5 for the proximal left anterior descending coronary artery (LAD) or left circumflex artery (LCX); 1.5 for the mid-LAD; and 1 for the distal LAD, the right coronary artery, or the mid-distal LCX. We further assessed whether concentrations of ox-HDL, ox-HDL/HDL-C, or relative intensity of ApoA-I glycation were associated with the Gensini score.

### Statistical analysis

Data are expressed as mean ± SD for continuous variables and as frequency and percentage for categorical variables. Comparisons between two groups were performed using two-tailed unpaired *t* tests or non-parametric Mann–Whitney tests. One-way ANOVA or the non-parametric Kruskal–Wallis test was used for multiple comparisons between ≥3 groups. Spearman’s correlation was used to calculate the correlations between plasma biomarker levels and the other measured parameters. The chi-square test or Fisher’s exact test was used for comparison of the qualitative data. GraphPad Prism 5.0 was used for statistical analysis. The significance level was set at *p* < 0.05.

### Ethics

The study was approved by the Human Ethics Committee of Union Hospital of Huazhong University of Science and Technology. Informed consent was obtained from all individual participants included in the study.

## Results

### Baseline characteristics

Age and gender were matched between the groups (Table 1). There were no significant differences in blood pressure or pulse rate between the groups. ApoA-I and LVEF were lower in patients with STEMI than that in the control group participants. BMI, LVEDD, total cholesterol, LDL-C, blood glucose, and HbA1c were higher in the STEMI group than in the controls. Blood glucose and HbA1c were significantly higher in the SH group than in the no SH group (Table 2). Gensini scores were lower in the HTB group than in the LTB group (Table 3).

**Table 1.** Clinical characteristics of patients in the STEMI and control group.

Characteristics	STEMI (*n* = 55)	Control (*n* = 48)	*p* value
Male, *n* (%)	49 (89.1)	36 (75.0)	0.0724
Age (years)	56.0 ± 11.6	56.2 ± 11.8	0.9488
BMI	25.7 ± 2.8	24.1 ± 2.5	0.0031
SBP	122.0 ± 23.7	125.8 ± 9.6	0.3021
DBP	75.8 ± 15.5	80.0 ± 6.6	0.0791
Pulse rate	80.4 ± 16.7	77.5 ± 13.6	0.3373
LVEF (%)	49.5 ± 9.5	58.9 ± 5.7	<0.0001
LVEDD (cm)	4.9 ± 0.6	4.2 ± 0.4	<0.0001
TC (mmol/L)	4.18 ± 1.01	3.42 ± 0.82	<0.0001
LDL-C (mmol/L)	2.48 ± 0.76	2.03 ± 0.43	0.0005
HDL-C (mmol/L)	1.10 ± 0.28	1.13 ± 0.31	0.5362
ApoA-I (g/L)	0.94 ± 0.22	1.12 ± 0.33	0.001
Creatinine (µmol/L)	86.83 ± 34.35	76.68 ± 17.20	0.0665
Blood glucose (mmol/L)	10.4 ± 4.3	4.9 ± 0.6	<0.0001
HbA1c (%)	6.32 ± 1.12	5.45 ± 0.55	<0.0001

Data are mean ± SD.

ApoA-I: apolipoprotein A-I; BMI: body mass index; DBP: diastolic blood pressure; HDL-C: high-density lipoprotein cholesterol; LDL-C: low-density lipoprotein cholesterol; LVEDD: left ventricular end-diastolic diameter; LVEF: left ventricular ejection fraction; SBP: systolic blood pressure; STEMI: ST-elevation myocardial infarction; TC: total cholesterol.

**Table 2.** Clinical characteristics of patients in the SH and no SH group.

Characteristics	SH (*n* = 28)	no SH (*n* = 27)	*p* values
Male, *n* (%)	25 (89.3)	24 (88.9)	1.0000
Age (years)	55.3 ± 10.9	56.7 ± 12.5	0.6543
BMI	25.3 ± 2.2	26.1 ± 3.3	0.3449
LVEF	50.5 ± 11.0	48.8 ± 8.5	0.5199
LDL-C (mmol/L)	2.48 ± 0.76	2.47 ± 0.76	0.9769
HDL-C (mmol/L)	1.07 ± 0.25	1.12 ± 0.31	0.5494
ApoA-1 (g/L)	0.94 ± 0.22	0.93 ± 0.23	0.8754
HbA1c (%)	6.76 ± 1.31	5.86 ± 0.64	0.002
Blood glucose (mmol/L)	13.2 ± 4.4	7.5 ± 1.1	<0.0001
Gensini score	59.72 ± 30.73	55.13 ± 19.42	0.5497
Culprit vessel, *n* (%)			
LAD	13 (46.4)	14 (51.9)	0.7896
LCX	3 (10.7)	4 (14.8)	0.7049
RCA	12 (42.9)	9 (33.3)	0.5815
History, *n* (%)			
Smoking	17 (60.7)	12 (44.4)	0.2847
Hypertension	17 (60.7)	15 (55.6)	0.7875
Diabetes	13 (46.4)	8 (29.6)	0.1755
Medications, *n* (%)			
β-blockers	14 (50.0)	11 (40.7)	0.5913
ACEI/ARB	10 (35.7)	17 (63.0)	0.0604
Statins	16 (57.1)	9 (33.3)	0.1060
Antiplatelet	12 (42.9)	17 (63.0)	0.1799
CCB	10 (35.7)	8 (29.6)	0.7753

Data are mean ± SD.

ACEI: angiotensin-converting enzyme inhibitors; ARB: angiotensin receptor blocker; CCB: calcium channel blockers; LAD: left anterior descending coronary artery; LCX: left circumflex artery; RCA: right coronary artery; SH: stress hyperglycaemia.

**Table 3.** Clinical characteristics of patients in the HTB and LTB group.

Characteristics	HTB (*n* = 26)	LTB (*n* = 29)	*p* value
Male, *n* (%)	23 (88.5)	26 (89.7)	1.0000
Age (years)	56.2 ± 11.6	55.9 ± 11.8	0.9172
BMI	25.5 ± 3.2	25.9 ± 2.5	0.5532
LVEF	50.5 ± 9.0	48.6 ± 10.1	0.4461
LDL-C (mmol/L)	2.31 ± 0.53	2.62 ± 0.90	0.1290
HDL-C (mmol/L)	1.09 ± 0.27	1.10 ± 0.29	0.9646
ApoA-1 (g/L)	0.91 ± 0.22	0.96 ± 0.22	0.4324
HbA1c (%)	6.12 ± 0.72	6.50 ± 1.37	0.2108
Blood glucose (mmol/L)	11.1 ± 2.8	9.8 ± 5.2	0.2819
Gensini score	50.02 ± 20.90	63.76 ± 28.06	0.0464
Culprit vessel, *n* (%)			
LAD	12 (46.2)	15 (51.7)	0.7891
LCX	3 (10.3)	4 (13.8)	0.6957
RCA	11 (42.3)	10 (34.5)	0.5890
History, *n* (%)			
Smoking	14 (53.8)	15 (51.7)	1.0000
Hypertension	15 (57.7)	17 (58.6)	1.0000
Diabetes	8 (30.7)	13 (44.8)	0.4052
Medications, *n* (%)			
β-blockers	10 (38.5)	15 (51.7)	0.4185
ACEI/ARB	11 (42.3)	16 (55.2)	0.4218
Statins	11 (42.3)	14 (48.3)	0.7876
Antiplatelet	13 (50)	16 (55.2)	0.7896
CCB	8 (30.8)	10 (34.5)	1.0000

Data are mean ± SD.

ACEI: angiotensin-converting enzyme inhibitors; ARB: angiotensin receptor blocker; CCB: calcium channel blockers; HTB: high thrombus burden; LAD: left anterior descending coronary artery; LCX: left circumflex artery; LTB: low thrombus burden; RCA: right coronary artery. ; BMI: body mass index; LVEF: left ventricular ejection fraction; HDL-C: high-density lipoprotein cholesterol; LDL-C: low-density lipoprotein cholesterol; ApoA-1: apolipoprotein A-I.

### Plasma ox-HDL concentrations and levels of gly-ApoA-I in the STEMI and control groups

**Figure 1.**
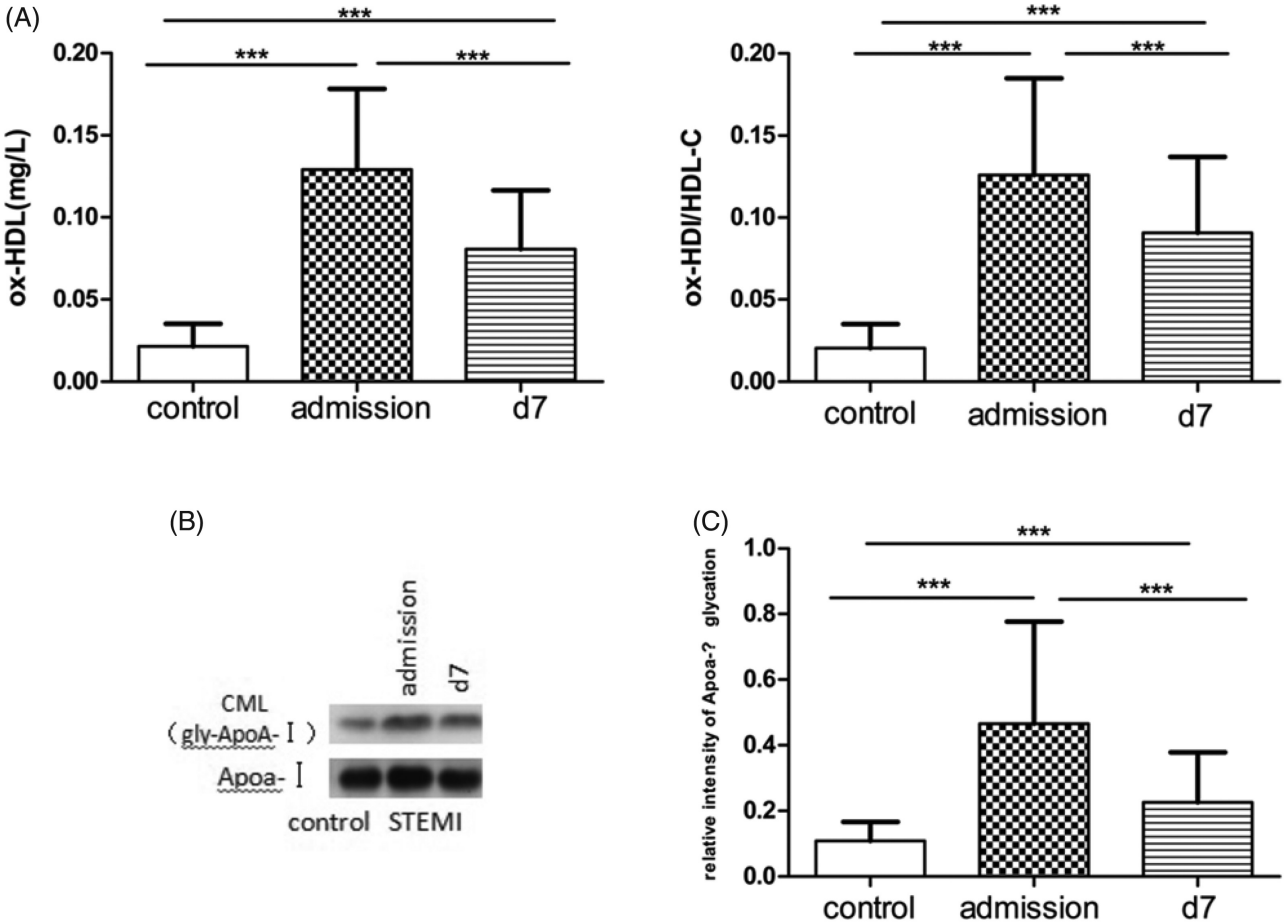
Plasma ox-HDL, ox-HDL/HDL-C, gly-ApoA-I, and relative intensity of ApoA-I glycation in STEMI patients. A: Plasma ox-HDL and ox-HDL/HDL-C were measured in STEMI patients on admission and d7. B: Gly-ApoA-I was determined in STEMI patients on admission and d7 by Western blot. CML: gly-ApoA-I. C: We also calculated relative intensity of ApoA-I glycation. #*p* > 0.05, *p < 0.05, **p < 0.01, and ***p < 0.001. Data are presented as mean ± SD.

Ox-HDL and gly-ApoA-I were measured on admission and d7 in the STEMI and control groups. Then, we calculated ox-HDL/HDL-C and the relative intensity of ApoA-I glycation. On admission, the levels of ox-HDL, ox-HDL/HDL-C, gly-ApoA-I, and relative intensity of ApoA-I glycation were significantly higher in the STEMI group than in the control group. At 7 days, these parameters declined; however, the values were also higher in the STEMI group than in the control group (Figure 1). In addition, there were no differences in the plasma concentrations of ox-HDL between patients with diabetes and those without diabetes (data not shown). However, the baseline level of ApoA-I glycation in patients with diabetes was higher than that of patients without diabetes (data not shown).

### Levels of ox-HDL, ox-HDL/HDL-C, gly-ApoA-I, and relative intensity of ApoA-I in the SH and no SH groups

**Figure 2.**
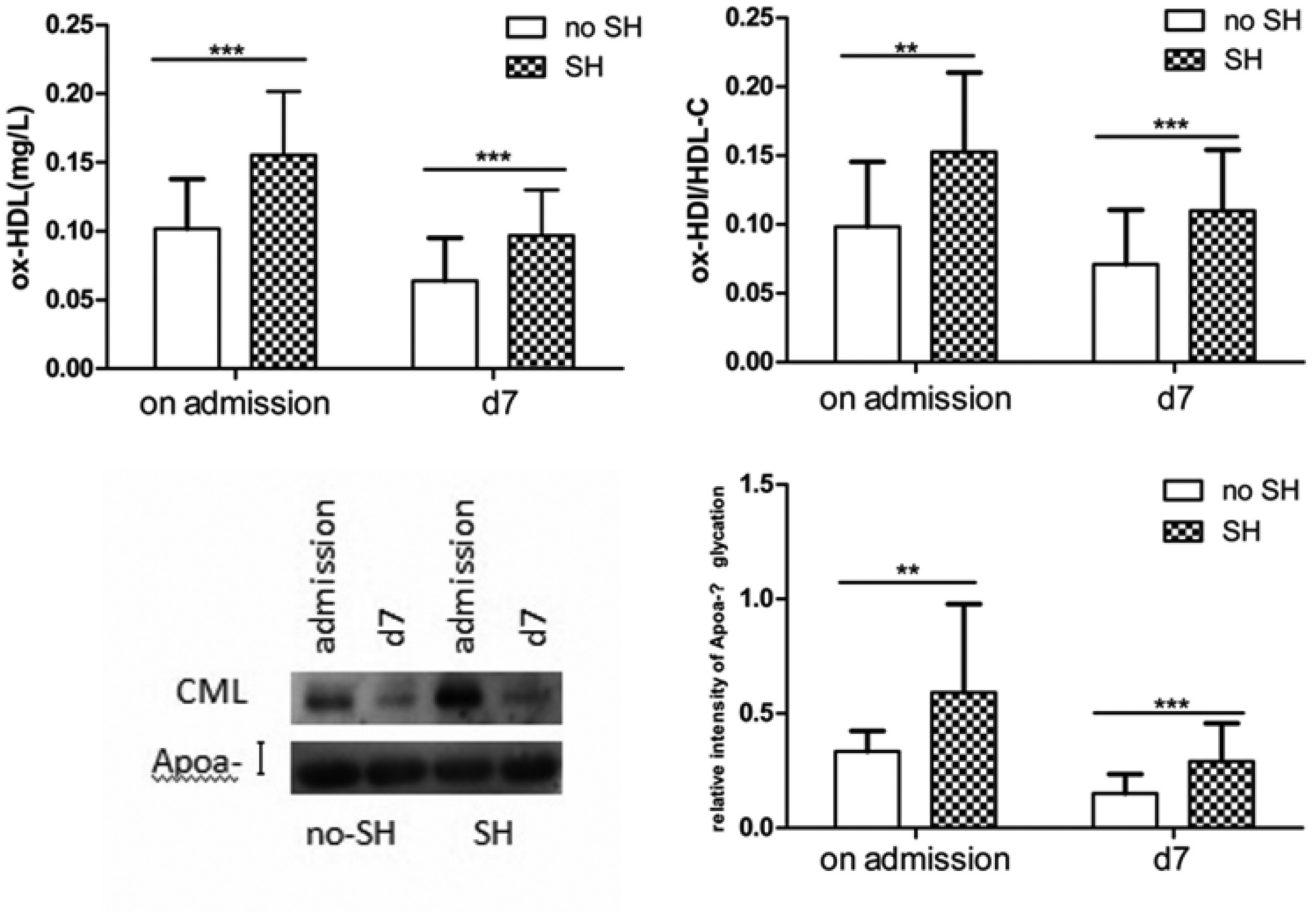
Plasma ox-HDL, ox-HDL/HDL-C, gly-ApoA-I, and relative intensity of ApoA-I glycation in the no SH group and SH group. Plasma ox-HDL, ox-HDL/HDL-C, gly-ApoA-I and relative intensity of ApoA-I glycation were quantified in the two groups (no SH group and SH group) on admission and d7. CML: gly-ApoA-I. #*p* > 0.05, *p < 0.05, **p < 0.01, and ***p < 0.001. Data are presented as mean ± SD.

Patients were divided into SH and no SH groups depending on the blood glucose concentration on admission. The levels of ox-HDL, ox-HDL/HDL-C, gly-ApoA-I, and the relative intensity of ApoA-I glycation were significantly higher in the SH group than in the no SH group both on admission and at d7 (Figure 2).

### Levels of ox-HDL, ox-HDL/HDL-C, gly-ApoA-I, and relative intensity of ApoA-I in the HTB and LTB groups

**Figure 3.**
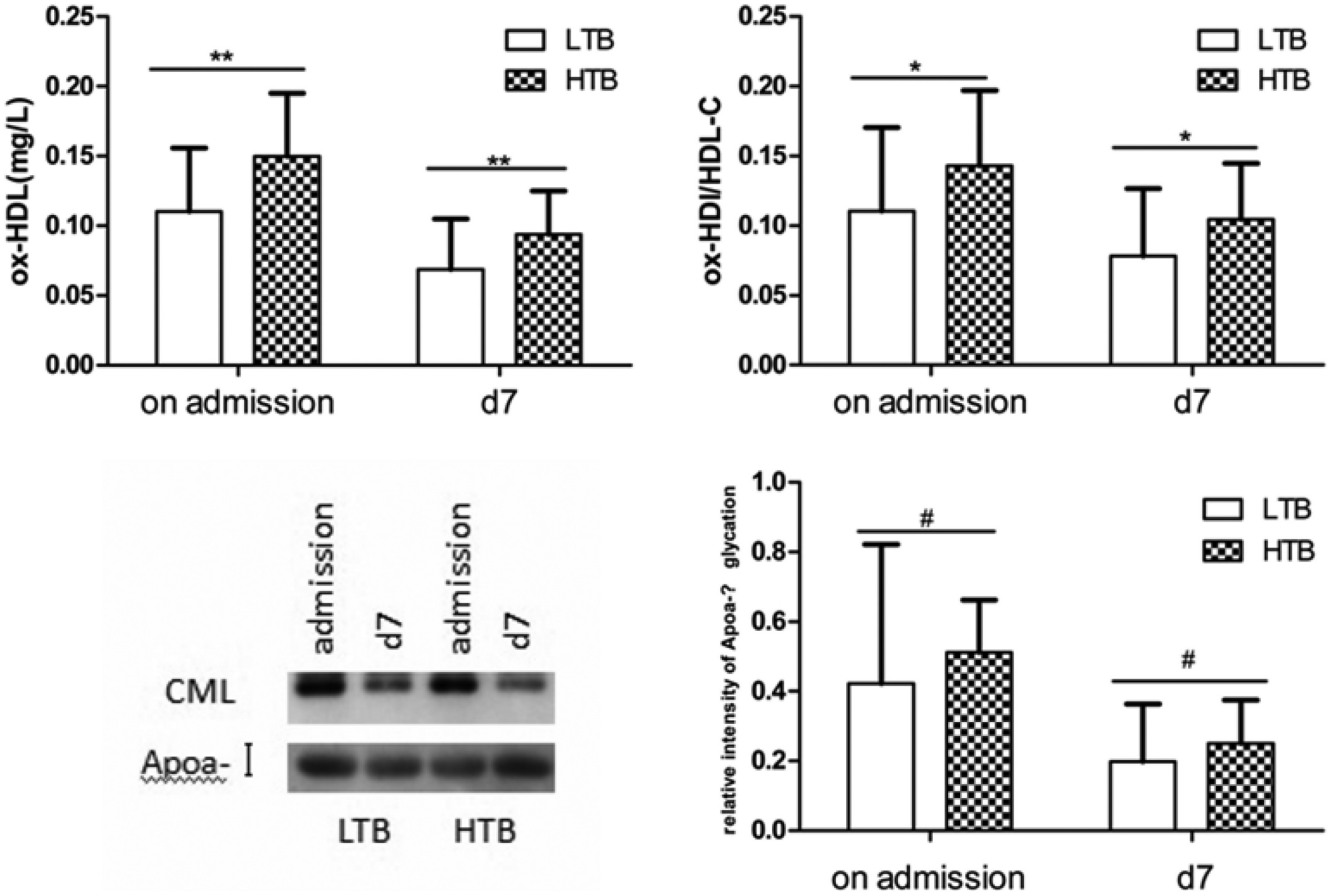
Plasma ox-HDL, ox-HDL/HDL-C, gly-ApoA-I, and relative intensity of ApoA-I glycation in the LTB group and HTB group. Plasma ox-HDL, ox-HDL/HDL-C, gly-ApoA-I, and relative intensity of ApoA-I glycation were quantified in the two groups (LTB group and HTB group) on admission and d7. CML: gly-ApoA-I. #*p* > 0.05, *p < 0.05, **p < 0.01, and ***p < 0.001. Data are presented as mean ± SD.

Patients were divided into a HTB and a LTB group depending on the thrombus burden. Levels of ox-HDL and ox-HDL/HDL-C were significantly higher in the HTB group than in the LTB group, both on admission and at d7 (Figure 3). However, gly-ApoA-I and relative intensity of ApoA-I glycation levels showed no significant differences between the HTB and LTB groups (Figure 3).

### Relationships between levels of ox-HDL, ox-HDL/HDL-C, and relative intensity of ApoA-I glycation and other measured parameters

**Figure 4.**
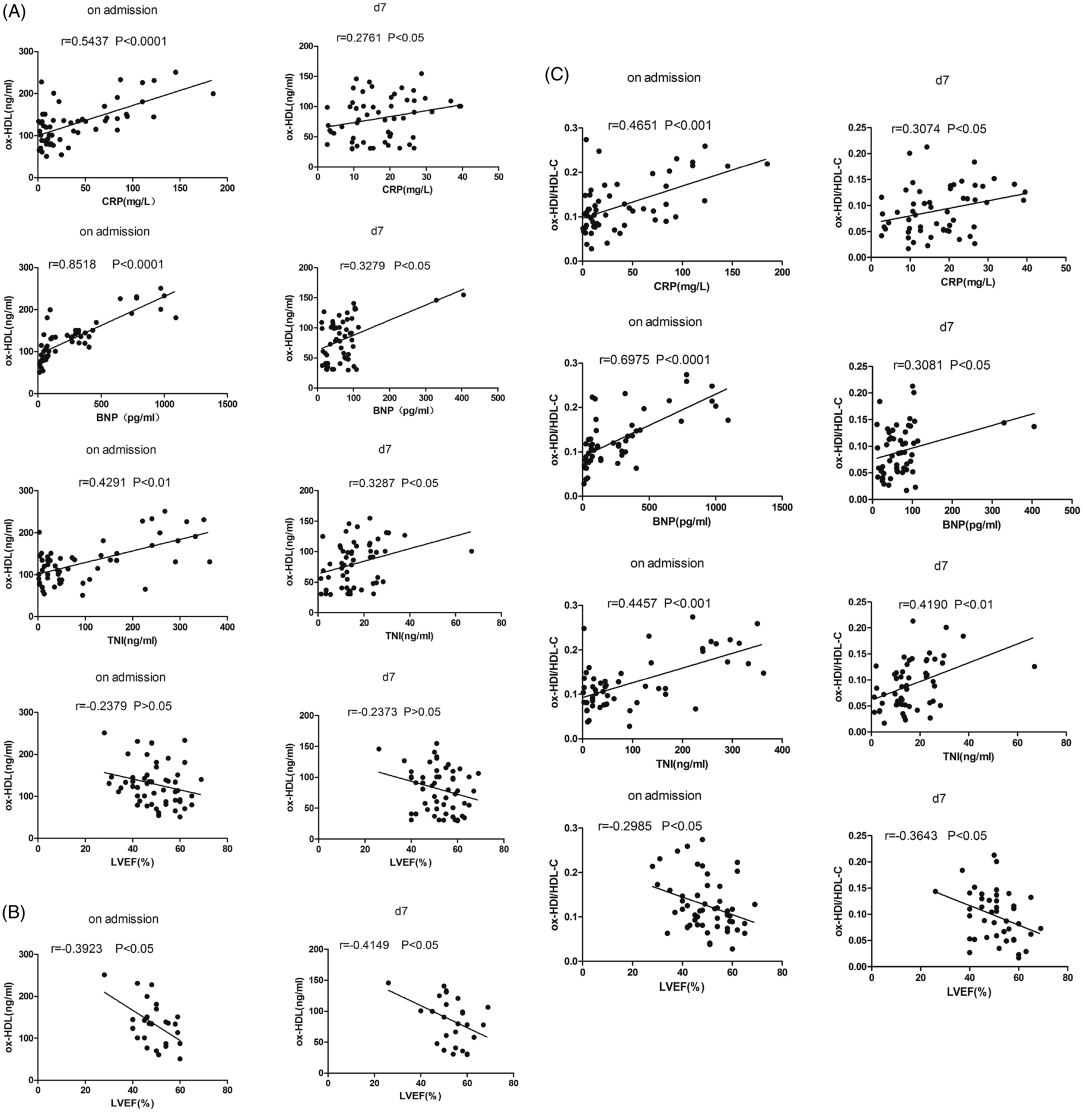
Spearman’s correlation analysis. (A) Plasma ox-HDL concentrations were positively correlated with CRP, BNP, and cTNI and were not correlated with LVEF in STEMI patients at each time point. (B) In patients whose culprit vessels were LAD, ox-HDL concentrations were negatively correlated with LVEF. (C) Ox-HDL/HDL-C levels were positively correlated with CRP, BNP, and cTNI and negatively correlated with LVEF at each time point. Data are presented as mean ± SD.

CRP, BNP, cTNI, and LVEF were also examined on admission and at d7 in the STEMI patients. We assessed whether the concentrations of ox-HDL, ox-HDL/HDL-C ratio, and relative intensity of ApoA-I glycation were associated with CRP, BNP, cTNI, and LVEF in patients with STEMI. It was found that that ox-HDL concentrations were positively correlated with CRP, BNP, and cTNI at each time point and were negatively correlated with LVEF in patients whose culprit vessels were LAD (Figure 4(A,B)). Ox-HDL/HDL-C levels were positively correlated with CRP, BNP, and cTNI and negatively correlated with LVEF in STEMI patients at each time point (Figure 4(C)). The relative intensity of ApoA-I glycation showed no relationship with CRP, BNP, cTNI, and LVEF (data not shown). We further assessed whether these markers were associated with the Gensini score that was used to quantify the severity of coronary artery stenosis in CAD. However, there were no significant correlations between these markers and the Gensini scores (data not shown).

## Discussion

We found that the concentrations of ox-HDL, ox-HDL/HDL-C, gly-ApoA-I, and the relative intensity of ApoA-I glycation were significantly higher in STEMI patients than in the controls on admission. These markers decreased at d7 but remained higher than the corresponding levels in the control group. Ox-HDL and gly-ApoA-I were higher in the SH group than in the no SH group at each time point. Patients with HTB had higher ox-HDL and ox-HDL/HDL-C levels than did the LTB group on admission and at d7. These results suggest that SH can lead to elevated levels of ox-HDL and gly-ApoA-I and therefore aggressive treatment is recommended; ox-HDL and ox-HDL/HDL-C were higher in the HTB group and may be used to quantify thrombus burden.

HDL protects against atherosclerosis through several mechanisms, including amelioration of endothelial dysfunction, removal of excess cholesterol from macrophages, as well as antioxidative, anti-inflammatory, and antiapoptotic effects (14–18). However, particles of HDL and/or its main protein constituent, ApoA-I, have diverse anti-atherosclerotic influences that are determined by their physicochemical properties (15). Oxidation and glycation modification in HDL/ApoA-I proteins and lipid constituents diminish the functions of HDL and may lead to CHD (6). Acute myocardial infarction (AMI) is a serious kind of CHD that is a major cause of morbidity and mortality worldwide (19). In the acute phase of STEMI, due to high oxidative stress and elevated blood glucose, HDL is more likely to undergo oxidation and glycation modification, forming a large amount of dysfunctional HDL. Ox-HDL and gly-ApoA-I might be new targets for study of myocardial infarction (MI).

SH is a common feature during the early phase after AMI, and it predicts poor outcomes (20,21). Several studies have demonstrated that hyperglycaemia has a direct detrimental effect on the ischaemic myocardium through many mechanisms, including increasing the release of inflammatory and vasoconstrictive factors that impair coronary endothelial function, contributing to the production of reactive oxygen species with consequent oxidative stress, and increasing platelet aggregation (12). This indicates that SH may lead to elevated ox-HDL and gly-ApoA-I levels. Although hyperglycaemia is usually managed with continuous insulin infusion, the optimal management of plasma glucose in STEMI patients with SH remains an open question. The current AHA/ACC guidelines recommend the use of an insulin-based regimen to achieve and maintain an average blood glucose concentration of less than 10 mmol/L (22). Our results point to the possibility to use a more aggressive glycaemic control in order to also reduce SH, if this might reduce HDL oxidation and glycation modification.

The major pathological conditions underlying STEMI are atherosclerosis, plaque rupture, and intracoronary thrombosis. Primary percutaneous coronary intervention (PCI) has become the preferred treatment in STEMI (23). The amount or ‘burden’ of thrombus in patients with STEMI undergoing primary PCI has been identified as a major determinant of outcomes, having been associated with reduced procedural success and worse early and late event-free survival (13,24–26). However, the few studies of thrombus burden grading systems did not use standardized criteria. Therefore, quantification of thrombus burden is an attractive tool because it enables identification of patients at risk of these complications. In addition, it precedes other angiographic reperfusion parameters and is particularly useful for the indication of thrombectomy and other adjuvant therapies.

The formation of arterial thrombosis is a result of the interplay of several factors, including vascular endothelial injury, platelet activation and aggregation, haemodynamic dysfunction, and qualitative and quantitative changes in coagulation or anticoagulation factors. It has been reported that HDL-C correlated with the angiographic presence of thrombus (27). HDL has the function of anti-inflammation, antioxidation, vascular endothelial protection, and antiplatelet action and coagulation. However, these functions are reduced when HDL is oxidized, indicating that ox-HDL may be correlated with the formation of arterial thrombosis. Our data showed that ox-HDL and ox-HDL/HDL-C were significantly higher in the HTB group than in the LTB group on admission and at d7. This indicates that ox-HDL and ox-HDL/HDL may be useful to measure and quantify thrombus burden.

In addition, we found that ox-HDL and ox-HDL/HDL-C levels positively correlated with CRP, BNP, and cTNI; the relative intensity of ApoA-I glycation had no relationship with CRP, BNP, or cTNI; and ox-HDL/HDL-C levels and LVEF were negatively correlated at each time point. More interestingly, ox-HDL negatively correlated with LVEF at each time point in patients whose culprit vessel was LAD. CRP, BNP, cTNI, and LVEF are common markers used in STEMI. A high CRP after AMI predicts infarct expansion, cardiac rupture, and mortality (28). The plasma concentration of BNP was highly related to MI size in patients with AMI and can be clinically used to evaluate MI size (29). cTNI has been extensively studied as a diagnostic and prognostic marker in ACS, and an increase in cTNI was highly indicative of myocardial injury (30). After STEMI, decreased left ventricular (LV) systolic function is significantly associated with poor clinical outcome (31). Our results suggest that circulating ox-HDL and ox-HDL/HDL-C are potentially novel biomarkers for patients with STEMI. An increasing number of studies have demonstrated that the levels of ox-HDL and gly-ApoA-I are increased in patients with AMI, and that HDL functions are impaired in these patients (7,8,32,33). Thus, ox-HDL and gly-ApoA-I might be new targets for the study of MI.

For years it was believed that HDL conferred protection against atherosclerosis and that elevated plasma HDL would reduce the risk of MI. However, the relevance of this concept has been challenged in recent years (34), and our study at least partially explained why elevated plasma HDL may not reduce the risk of MI.

Our study also has several limitations. First, the sample size was not very large. It is difficult for patients to stay in the hospital for more than 7 days. Second, our patients were younger and thinner than the patients in a couple of European or American studies of a similar kind, and there were very few female patients. This may be attributed to: (1) Asians having smaller body sizes than Europeans or Americans; (2) the earlier age of onset of STEMI in China; (3) the incidence of STEMI being higher in men than in women; and (4) again, our sample size being small. Third, approximately 40% of the STEMI patients were diabetic. We measured the plasma concentrations of ox-HDL in STEMI patients and found no significant difference between patients with diabetes and those without diabetes (data not shown). However, the baseline level of ApoA-I glycation in patients with diabetes was higher than in those without diabetes, which is one of the reasons for the inverse relationship between the relative intensity of ApoA-I glycation and CRP, BNP, cTNI, and LVEF. In future studies of the issues related to ApoA-I glycation, we may only select STEMI patients with diabetes or without diabetes as research subjects. Fourth, anti–N´-(carboxymethyl)-lysine (CML) can also be formed through oxidative reaction and inflammation (35). Thus, the relative intensity of ApoA-I glycation in our study may have appeared higher than it actually was. Fifth, we were not able to explain the mechanism and exact role of ox-HDL and gly-ApoA-I on STEMI. These mechanisms should be further studied in animal models.

## Conclusions

This small study in patients with STEMI indicates that SH may be associated with elevated levels of ox-HDL and gly-ApoA-I, and that the degree of thrombus burden may be associated with ox-HDL and ox-HDL/HDL. Further studies will be needed to verify the usefulness of these variables in MI patients.
